# Characteristics and Clinical Outcomes of Children and Adolescents Aged <18 Years Hospitalized with COVID-19 — Six Hospitals, United States, July–August 2021

**DOI:** 10.15585/mmwr.mm705152a3

**Published:** 2021-12-31

**Authors:** Valentine Wanga, Megan E. Gerdes, Dallas S. Shi, Rewa Choudhary, Theresa M. Dulski, Sophia Hsu, Osatohamwen I. Idubor, Bryant J. Webber, Arthur M. Wendel, Nickolas T. Agathis, Kristi Anderson, Tricia Boyles, Sophia K. Chiu, Eleanor S. Click, Juliana Da Silva, Hannah Dupont, Mary Evans, Jeremy A.W. Gold, Julia Haston, Pamela Logan, Susan A. Maloney, Marisol Martinez, Pavithra Natarajan, BMBS, Kevin B. Spicer, Mark Swancutt, Valerie A. Stevens, Jessica Brown, Gyan Chandra, Megan Light, Frederick E. Barr, Jessica Snowden, Larry K. Kociolek, Matthew McHugh, David Wessel, Joelle N. Simpson, Kathleen C. Gorman, Kristen A. Breslin, Roberta L. DeBiasi, Aaron Thompson, Mark W. Kline, Julie A. Bloom, Ila R. Singh, Michael Dowlin, Mark Wietecha, Beth Schweitzer, Sapna Bamrah Morris, Emily H. Koumans, Jean Y. Ko, Anne A. Kimball, David A. Siegel

**Affiliations:** ^1^CDC COVID-19 Response Team; ^2^Epidemic Intelligence Service, CDC; ^3^Arkansas Children’s, Little Rock, Arkansas; ^4^Ann & Robert H. Lurie Children's Hospital of Chicago, Chicago, Illinois; ^5^Children’s National Hospital, Washington, DC; ^6^Children’s Hospital New Orleans, New Orleans, Louisiana; ^7^Tulane University School of Medicine and LSU Health, New Orleans, Louisiana; ^8^Department of Pediatrics, Baylor College of Medicine, Houston, Texas; ^9^Department of Pathology and Immunology, Baylor College of Medicine, Houston, Texas; ^10^Children’s Hospital Association, Washington, DC.

During June 2021, the highly transmissible^†^ B.1.617.2 (Delta) variant of SARS-CoV-2, the virus that causes COVID-19, became the predominant circulating strain in the United States. U.S. pediatric COVID-19–related hospitalizations increased during July–August 2021 following emergence of the Delta variant and peaked in September 2021.^§^ As of May 12, 2021, CDC recommended COVID-19 vaccinations for persons aged ≥12 years,^¶^ and on November 2, 2021, COVID-19 vaccinations were recommended for persons aged 5–11 years.** To date, clinical signs and symptoms, illness course, and factors contributing to hospitalizations during the period of Delta predominance have not been well described in pediatric patients. CDC partnered with six children’s hospitals to review medical record data for patients aged <18 years with COVID-19–related hospitalizations during July–August 2021.^††^ Among 915 patients identified, 713 (77.9%) were hospitalized for COVID-19 (acute COVID-19 as the primary or contributing reason for hospitalization), 177 (19.3%) had incidental positive SARS-CoV-2 test results (asymptomatic or mild infection unrelated to the reason for hospitalization), and 25 (2.7%) had multisystem inflammatory syndrome in children (MIS-C), a rare but serious inflammatory condition associated with COVID-19.^§§^ Among the 713 patients hospitalized for COVID-19, 24.7% were aged <1 year, 17.1% were aged 1–4 years, 20.1% were aged 5–11 years, and 38.1% were aged 12–17 years. Approximately two thirds of patients (67.5%) had one or more underlying medical conditions, with obesity being the most common (32.4%); among patients aged 12–17 years, 61.4% had obesity. Among patients hospitalized for COVID-19, 15.8% had a viral coinfection^¶¶^ (66.4% of whom had respiratory syncytial virus [RSV] infection). Approximately one third (33.9%) of patients aged <5 years hospitalized for COVID-19 had a viral coinfection. Among 272 vaccine-eligible (aged 12–17 years) patients hospitalized for COVID-19, one (0.4%) was fully vaccinated.*** Approximately one half (54.0%) of patients hospitalized for COVID-19 received oxygen support, 29.5% were admitted to the intensive care unit (ICU), and 1.5% died; of those requiring respiratory support, 14.5% required invasive mechanical ventilation (IMV). Among pediatric patients with COVID-19–related hospitalizations, many had severe illness and viral coinfections, and few vaccine-eligible patients hospitalized for COVID-19 were vaccinated, highlighting the importance of vaccination for those aged ≥5 years and other prevention strategies to protect children and adolescents from COVID-19, particularly those with underlying medical conditions.

Data were collected from six U.S. children’s hospitals located in areas with high COVID-19 incidence during July–August 2021 (Arkansas, District of Columbia, Florida, Illinois, Louisiana, and Texas).^†††^ Data from hospitalized patients aged <18 years with COVID-19 or SARS-CoV-2 infection^§§§^ were abstracted from electronic medical records using REDCap software (version 11.1.8; Vanderbilt University). Patients were categorized^¶¶¶^ by reason for hospitalization: 1) acute COVID-19, 2) incidental positive SARS-CoV-2 test result, or 3) MIS-C. Patient demographic characteristics, medical history, coinfections, and disease severity, including need for and duration of respiratory support, ICU admission, IMV, extracorporeal membrane oxygenation (ECMO),**** and deaths were abstracted from the medical record. Among patients hospitalized for COVID-19, presence of underlying medical conditions (including obesity),^††††^ viral coinfection, and illness course were described by age group. Pearson’s chi-square and Kruskal-Wallis tests were used to compare categorical and continuous variables, respectively; p*-*values <0.05 were considered statistically significant. All analyses were conducted using SAS (version 9.4; SAS Institute) and R (Version 4.0.3; R Foundation for Statistical Computing). This activity was reviewed by CDC and the other participating institutions and was conducted consistent with applicable federal law and CDC policy.^§§§§^

Among 915 patients aged <18 years, 713 (77.9%) were hospitalized for COVID-19, 177 (19.3%) had incidental SARS-CoV-2 infections, and 25 (2.7%) had MIS-C (Table 1). Among all 915 patients, 22.5% were aged <1 year, 18.3% were aged 1–4 years, 21.5% were aged 5–11 years, and 37.7% were aged 12–17 years. Among the 713 patients hospitalized for COVID-19, approximately one half (373; 52.3%) were male, 210 (29.5%) were non-Hispanic White persons, 202 (28.3%) were non-Hispanic Black persons or African American persons (Black), and 211 (29.6%) were Hispanic persons.

**TABLE 1:** Demographic characteristics and COVID-19 vaccination status of hospitalized patients aged <18 years with a positive SARS-CoV-2 test result or diagnosis of COVID-19, by reason for hospitalization — six hospitals,* United States, July–August 2021

Characteristic	Reason for hospitalization, no. (%)			
	Overall	COVID-19^†^	Incidental positive SARS-CoV-2 test result	MIS-C^§^
	N = 915 (100)	n = 713 (77.9)	n = 177 (19.3)	n = 25 (2.7)
**Age, yrs, median (IQR)**	8.0 (1.3-14.0)	8.0 (1.0-14.0)	9.0 (2.0-14.0)	8.0 (4.0-13.0)
**Age group, yrs**				
<1	206 (22.5)	176 (24.7)	29 (16.4)	1 (4.0)
1–4	167 (18.3)	122 (17.1)	36 (20.3)	9 (36.0)
5–11	197 (21.5)	143 (20.1)	47 (26.6)	7 (28.0)
12–17	345 (37.7)	272 (38.1)	65 (36.7)	8 (32.0)
**Sex**				
Female	437 (47.8)	340 (47.7)	87 (49.2)	10 (40.0)
Male	478 (52.2)	373 (52.3)	90 (50.8)	15 (60.0)
**Race/Ethnicity**				
White, non-Hispanic	277 (30.3)	210 (29.5)	59 (33.3)	8 (32.0)
Black or African American, non-Hispanic	260 (28.4)	202 (28.3)	48 (27.1)	10 (40.0)
Hispanic	267 (29.2)	211 (29.6)	52 (29.4)	4 (16.0)
Other, Non-Hispanic^¶^	42 (4.6)	35 (4.9)	6 (3.4)	1 (4.0)
Unknown	69 (7.5)	55 (7.7)	12 (6.8)	2 (8.0)
**COVID-19 vaccination status**				
Eligible for vaccination (aged 12–17 yrs)**	345 (37.7)	272 (38.1)	65 (36.7)	8 (32.0)
Fully vaccinated	3 (0.9)	1 (0.4)	2 (3.1)	0 (—)
Partially vaccinated	18 (5.2)	12 (4.4)	4 (6.2)	2 (25.0)
Not vaccinated	224 (64.9)	196 (72.1)	22 (33.8)	6 (75.0)
Unknown vaccination status	100 (29.0)	63 (23.2)	37 (56.9)	0 (—)
Ineligible for vaccination (aged <12 yrs)	570 (62.3)	441 (61.9)	112 (63.3)	17 (68.0)

**Abbreviation:** MIS-C = multisystem inflammatory syndrome in children.

* The six children’s hospitals were in Arkansas, District of Columbia, Florida, Illinois, Louisiana, and Texas.

^†^ Patients hospitalized for COVID-19 included patients with acute COVID-19 as the primary reason for hospitalization or with acute COVID-19 as a secondary or contributing reason for hospitalization, based on chart review.

^§^ Patients with MIS-C as the reason for hospitalization included patients who met the clinical case definition for MIS-C (clinically severe illness requiring hospitalization in a person aged <21 years with fever, laboratory evidence of inflammation, multisystem [≥2] organ involvement and no alternative plausible diagnosis, and evidence of current or recent SARS-CoV-2 infection by reverse transcription polymerase chain reaction, serology or antigen test, or COVID-19 exposure within the 4 weeks preceding symptom onset [https://emergency.cdc.gov/han/2020/han00432.asp]) and were hospitalized for diagnosis and management of MIS-C, based on chart review.

^¶^ Other race/ethnicity includes Asian, Native Hawaiian or Other Pacific Islander, American Indian or Alaska Native, and Other (not specified).

** Fully vaccinated was defined as having received 2 doses of an mRNA-based COVID-19 vaccine ≥14 days before the hospital admission date. Partially vaccinated was defined as having received only 1 dose of an mRNA-based COVID-19 vaccine ≥14 days before hospitalization. All vaccinated patients in this study received the Pfizer-BioNTech (BNT162b2) vaccine.

Among the 713 patients hospitalized for COVID-19, 32.5%, 51.3%, and 16.1% had zero, one or two, and three or more underlying medical conditions, respectively (Table 2). The most common conditions were obesity (32.4%), asthma or reactive airway disease (16.0%), and feeding tube dependence (8.3%). Among patients aged 12–17 years, 61.4% had obesity (60.5% of whom had severe obesity). Among patients aged 5–11 years, 33.6% had obesity (60.4% of whom had severe obesity). Among patients hospitalized for COVID-19, 210 (29.5%) had ICU admissions, eight (1.1%) received ECMO, and 11 (1.5%) died. Of the 385 (54.0%) patients hospitalized for COVID-19 who received oxygen support, high-flow nasal cannula was the most common highest level of support (142; 36.9%); 56 (14.5%) patients received IMV. Across all age groups, the median hospital stay was 3 days, and the median IMV duration was 7 days. Patients aged 12–17 years had the longest median hospitalizations (4 days) and IMV requirement (9.5 days). Viral coinfection was common among patients aged <1 year (32.4%) and 1–4 years (36.1%); overall, approximately two thirds of viral coinfections were with RSV (Table 2).

**TABLE 2:** Hospitalization and illness course among children and adolescents aged <18 years hospitalized for COVID-19,* by age group — six hospitals,^†^ United States, July–August 2021

Characteristic	Age group, yrs, no. (%)					
	Overall	<1	1–4	5–11	12–17	p-value^§^
	(N = 713)	(n = 176)	(n = 122)	(n = 143)	(n = 272)	
**No. of underlying medical conditions**						
None	232 (32.5)	124 (70.5)	51 (41.8)	25 (17.5)	32 (11.8)	<0.001
1–2	366 (51.3)	47 (26.7)	46 (37.7)	89 (62.2)	184 (67.6)	
≥3	115 (16.1)	5 (2.8)	25 (20.5)	29 (20.3)	56 (20.6)	
**Five most prevalent conditions by system**						
Metabolic or endocrine^¶^	258 (36.2)	2 (1.1)	17 (13.9)	59 (41.3)	180 (66.2)	<0.001
Obesity**	231 (32.4)	—	16 (13.1)	48 (33.6)	167 (61.4)	
Obesity	90 (39.0)	—	14 (87.5)	17 (35.4)	59 (35.3)	<0.001
Severe obesity	131 (56.7)	—	1 (6.3)	29 (60.4)	101 (60.5)	
Obesity, unknown severity	10 (4.3)	—	1 (6.3)	2 (4.2)	7 (4.2)	
Neurologic or developmental^††^	144 (20.2)	41 (23.3)	33 (27.0)	28 (19.6)	42 (15.4)	0.038
Seizure disorder	57 (8.0)	6 (3.4)	15 (12.3)	14 (9.8)	22 (8.1)	0.033
Respiratory^§§^	142 (19.9)	7 (4.0)	18 (14.8)	34 (23.8)	83 (30.5)	<0.001
Asthma or RAD	114 (16.0)	2 (1.1)	12 (9.8)	26 (18.2)	74 (27.2)	<0.001
Gastrointestinal or hepatic^¶¶^	85 (11.9)	12 (6.8)	28 (23.0)	16 (11.2)	29 (10.7)	<0.001
Feeding tube dependent	59 (8.3)	7 (4.0)	23 (18.9)	13 (9.1)	16 (5.9)	<0.001
Psychiatric***	58 (8.1)	0 (—)	0 (—)	13 (9.1)	45 (16.5)	<0.001
Depression	23 (3.2)	0 (—)	0 (—)	1 (0.7)	22 (8.1)	<0.001
**Multiple admissions**						
Yes	28 (3.9)	4 (2.3)	5 (4.1)	6 (4.2)	13 (4.8)	0.607
No	685 (96.1)	172 (97.7)	117 (95.9)	137 (95.8)	259 (95.2)	
Unknown	14 (2.4)	3 (1.9)	2 (2.0)	2 (1.9)	6 (2.7)	
Hospital length of stay, median days (IQR)	3.0 (1.0–7.0)	3.0 (1.0–6.8)	3.0 (2.0–4.5)	3.0 (1.0–7.0)	4.0 (2.0–8.0)	0.187
**Admitted to ICU**						
Yes	210 (29.5)	34 (19.3)	31 (25.4)	37 (25.9)	108 (39.7)	<0.001
No	503 (70.5)	142 (80.7)	91 (74.6)	106 (74.1)	164 (60.3)	
Total length of stay in ICU, median days (IQR)	3.0 (1.0–7.0)	3.0 (1.0–6.8)	3.0 (2.0–4.5)	3.0 (1.0–7.0)	4.0 (2.0–8.0)	0.187
**Highest level of respiratory support required**						
No oxygen support	328 (46.0)	94 (53.4)	57 (46.7)	82 (57.3)	95 (34.9)	<0.001
Oxygen support	385 (54.0)	82 (46.6)	65 (53.3)	61 (42.7)	177 (65.1)	
Nasal cannula	111 (28.8)	22 (26.8)	14 (21.5)	24 (39.3)	51 (28.8)	
Mask	7 (1.8)	0 (—)	2 (3.1)	1 (1.6)	4 (2.3)	
CPAP or BiPAP	69 (17.9)	5 (6.1)	10 (15.4)	11 (18.0)	43 (24.3)	
High-flow nasal cannula	142 (36.9)	43 (52.4)	32 (49.2)	14 (23.0)	53 (29.9)	
IMV	56 (14.5)	12 (14.6)	7 (10.8)	11 (18.0)	26 (14.7)	
Duration on IMV, median days (IQR)	7.0 (4.0–14.0)	6.0 (4.8–12.3)	6.0 (2.0–11.5)	5.5 (1.8–10.3)	9.5 (5.0–21.3)	0.596
**ECMO required**						
Yes	8 (1.1)	1 (0.6)	1 (0.8)	1 (0.7)	5 (1.8)	0.567
No	705 (98.9)	175 (99.4)	121 (99.2)	142 (99.3)	267 (98.2)	
Duration on ECMO, median days (IQR)	12.0 (5.5–17.8)	1.0 (1.0–1.0)	13.0 (13.0–13.0)	—	15.0 (11.0–26.0)	0.247
**Viral coinfection^†††^**	113 (15.8)	57 (32.4)	44 (36.1)	6 (4.2)	6 (2.2)	<0.001
RSV	75 (66.4)	42 (73.7)	26 (59.1)	4 (66.7)	3 (50.0)	<0.001
**Discharge status**						
Discharged alive	702 (98.5)	174 (98.9)	122 (100.0)	142 (99.3)	264 (97.1)	0.231
Deceased	11 (1.5)	2 (1.1)	0 (—)	1 (0.7)	8 (2.9)	

**Abbreviations:** BiPAP = bilevel positive airway pressure; BMI = body mass index; CPAP = continuous positive airway pressure; ECMO = extracorporeal membrane oxygenation; ICU = intensive care unit; IMV = invasive mechanical ventilation; RAD = reactive airway disease; RSV = respiratory syncytial virus.

* Patients hospitalized for COVID-19 included patients with acute COVID-19 as the primary reason for hospitalization or with acute COVID-19 as a secondary or contributing reason for hospitalization, based on chart review.

^†^ The six children’s hospitals were in Arkansas, District of Columbia, Florida, Illinois, Louisiana, and Texas.

^§^ Clinical characteristics and outcomes were compared among groups using Pearson’s chi-square test for categorical variables and a Kruskal-Wallis test for nonnormally distributed variables.

^¶^ Metabolic and endocrine conditions included dyslipidemia, obesity, thyroid disorder, type 1 diabetes, type 2 diabetes, and other endocrine disorders.

** For children aged ≥2 years, height and weight were used to calculate BMI (kg/m^2^). BMI percentiles were calculated using BMI, age, and sex. Those children with BMI percentiles ≥95th percentile were considered to have obesity and those with BMI ≥120% of the 95th percentile were considered to have severe obesity. BMI data extracted from charts were used if height or weight was missing. If BMI was missing or unable to be calculated, a diagnosis of obesity recorded in charts was used and severity of obesity was unable to be assessed. Obesity was not assessed for children aged <2 years.

^††^ Neurologic and developmental conditions included attention deficit hyperactivity disorder, autism spectrum disorder, cerebral palsy, cognitive disfunction, muscular dystrophy, neural tube defect or spina bifida, neurologic or neurodevelopmental disorder, neuropathy, plegias or paralysis, preterm birth (for children aged <2 years only), seizure disorder, and wheelchair/walker-dependence or bed-bound status.

^§§^ Respiratory conditions included active tuberculosis, asthma or reactive airway disease, chronic hypoxemic respiratory failure with oxygen or ventilator dependence, cystic fibrosis, current smoking or e-cigarette use, tracheostomy dependence, and other chronic lung diseases.

^¶¶^ Gastrointestinal or hepatic conditions included Crohn's disease, feeding tube dependence, liver disease, malnutrition, ulcerative colitis, and other gastrointestinal disorders.

*** Psychiatric conditions included anxiety, borderline personality disorder, depression, substance use disorder, and other psychiatric diagnoses.

^†††^ Patients were considered to have a viral coinfection if they had ≥1 of the following infections: type A influenza, type B influenza, unspecified influenza, coronavirus 229e, coronavirus hku1, coronavirus nl63, coronavirus 0c43, respiratory syncytial virus, adenovirus, parainfluenza type 1, parainfluenza type 2, parainfluenza type 3, parainfluenza type 4, human metapneumovirus, rhinovirus enterovirus, or other viral coinfection.

Among 272 vaccine-eligible patients hospitalized for COVID-19, one (0.4%) was fully vaccinated and 12 (4.4%) were partially vaccinated with an mRNA COVID-19 vaccine at the time of hospitalization (Table 1).

A higher percentage of patients hospitalized for COVID-19 with any underlying condition were admitted to the ICU (34.7%) compared with those without an underlying condition (18.5%) (p<0.001) (Table 3). The duration of hospitalization was longer for patients with obesity (median = 4 days [IQR = 2.0–7.5 days]) than that for those without obesity (median = 2 days [IQR = 1.0–5.0 days]) (p<0.001). A higher proportion of patients with obesity were admitted to the ICU (41.1%) than were those without obesity (23.9%) (p<0.001). A higher proportion of patients with viral coinfection required oxygen support (69.0%) compared with those without viral coinfection (51.2%) (p<0.001).

**TABLE 3:** Hospitalization and illness course among children and adolescents aged <18 years hospitalized for COVID-19* by presence of underlying medical conditions, obesity, and viral coinfection — six hospitals,^†^ United States, July–August 2021

Characteristic	No. (%)								
	Underlying medical condition			Obesity^§^			Viral coinfection		
	Yes (n = 481)	No (n = 232)	p-value^¶^	Yes (n = 231)	No (n = 482)	p-value^¶^	Yes (n = 113)	No (n = 600)	p-value^¶^
**Multiple admissions**									
Yes	23 (4.8)	5 (2.2)	0.137	12 (5.2)	16 (3.3)	0.317	3 (2.7)	25 (4.2)	0.621
No	458 (95.2)	227 (97.8)		219 (94.8)	466 (96.7)		110 (97.3)	575 (95.8)	
Hospital length of stay, median days (IQR)	3.0 (2.0–7.0)	2.0 (1.0–4.0)	<0.001	4.0 (2.0–7.5)	2.0 (1.0–5.0)	<0.001	3.0 (2.0–6.0)	3.0 (1.0–6.0)	0.085
**Admitted to ICU**									
Yes	167 (34.7)	43 (18.5)	<0.001	95 (41.1)	115 (23.9)	<0.001	36 (31.9)	174 (29.0)	0.618
No	314 (65.3)	189 (81.5)		136 (58.9)	367 (76.1)		77 (68.1)	426 (71.0)	
ICU length of stay, median days (IQR)	4.0 (1.0–8.0)	2.0 (1.0–4.0)	0.023	4.0 (2.0–8.0)	3.0 (1.0–6.5)	0.014	4.0 (1.8–10.3)	3.0 (1.0–7.0)	0.37
**Highest level of respiratory support required**									
None	199 (41.4)	129 (55.6)	<0.001	61 (26.4)	267 (55.4)	<0.001	35 (31.0)	293 (48.8)	<0.001
Oxygen support	282 (58.6)	103 (44.4)		170 (73.6)	215 (44.6)		78 (69.0)	307 (51.2)	
Nasal cannula	77 (27.3)	34 (33.0)		47 (27.6)	64 (29.8)		10 (12.8)	101 (32.9)	
Mask	6 (2.1)	1 (1.0)		2 (1.2)	5 (2.3)		1 (1.3)	6 (2.0)	
CPAP or BIPAP	62 (22.0)	7 (6.8)		43 (25.3)	26 (12.1)		8 (10.3)	61 (20.0)	
High-flow nasal cannula	91 (32.3)	51 (49.5)		55 (32.4)	87 (40.5)		46 (59.0)	96 (31.3)	
IMV	46 (16.3)	10 (9.7)		23 (13.5)	33 (15.3)		13 (16.7)	43 (14.0)	
IMV duration, median days (IQR)	8.0 (4.0–15.0)	5.5 (1.0–6.8)	0.161	8.0 (5.0–14.5)	6.0 (3.8–13.5)	0.472	6.0 (5.0–13.0)	7.0 (3.0–14.5)	0.804
**ECMO required**									
Yes	5 (1.0)	3 (1.3)	1.000	5 (2.2)	3 (0.6)	0.147	2 (1.8)	6 (1.0)	0.821
No	476 (99.0)	229 (98.7)		226 (97.8)	479 (99.4)		111 (98.2)	594 (99.0)	
ECMO duration, median days (IQR)	15.0 (11.0–26.0)	1.0 (0.5–7.0)	0.101	15.0 (11.0–26.0)	1.0 (0.5–7.0)	0.101	7.0 (4.0–10.0)	13.0 (8.0–23.3)	0.505
Viral coinfection**	49 (10.2)	64 (27.6)	<0.001	7 (3.0)	106 (22.0)	<0.001	113 (100.0)	0 (—)	<0.001
RSV	31 (63.3)	44 (68.8)	<0.001	2 (28.6)	73 (68.9)	<0.001	75 (66.4)	0 (—)	<0.001
**Discharge status**									
Discharged alive	472 (98.1)	230 (99.1)	0.517	227 (98.3)	475 (98.5)	0.595	111 (98.2)	591 (98.5)	0.81
Deceased	9 (1.9)	2 (0.9)		4 (1.7)	7 (1.5)		2 (1.8)	9 (1.5)	

**Abbreviations:** BIPAP = bilevel positive airway pressure; BMI = body mass index; CPAP = continuous positive airway pressure; ECMO = extracorporeal membrane oxygenation; ICU = intensive care unit; IMV = invasive mechanical ventilation; RSV = respiratory syncytial virus.

* Patients hospitalized for COVID-19 included patients with acute COVID-19 as the primary reason for hospitalization or with acute COVID-19 as a secondary or contributing reason for hospitalization, based on chart review.

^†^ The six children’s hospitals were in Arkansas, District of Columbia, Florida, Illinois, Louisiana, and Texas.

^§^ For children aged ≥2 years, height and weight were used to calculate BMI (kg/m^2^). BMI percentiles were calculated using BMI, age, and sex. Those children with BMI percentiles ≥95th percentile were considered to have obesity, and those with BMI ≥120% of the 95th percentile were considered to have severe obesity. BMI data extracted from charts were used if height or weight was missing. If BMI was missing or unable to be calculated, a diagnosis of obesity recorded in charts was used and severity of obesity was unable to be assessed. Obesity was not assessed for children aged <2 years.

^¶^ Clinical characteristics and outcomes were compared among groups using Pearson’s chi-square test for categorical variables and a Kruskal-Wallis test for nonnormally distributed variables.

** Patients were considered to have a viral coinfection if they had ≥1 of the following infections: type A influenza, type B influenza, unspecified influenza, coronavirus 229e, coronavirus hku1, coronavirus nl63, coronavirus 0c43, respiratory syncytial virus, adenovirus, parainfluenza type 1, parainfluenza type 2, parainfluenza type 3, parainfluenza type 4, human metapneumovirus, rhinovirus, enterovirus, or other viral coinfection.

## Discussion

In this study of six U.S. hospitals during July–August, 2021, approximately three quarters of pediatric patients with COVID-19–related hospitalizations were hospitalized for COVID-19. The majority of those hospitalized for COVID-19 were Black or Hispanic and were aged <5 or 12–17 years. Approximately one third of patients aged <1 and 1–4 years had a viral coinfection, approximately one third of patients aged 5–11 years and approximately two thirds of patients aged 12–17 years had obesity. Less than 1% of vaccine-eligible patients were fully vaccinated against COVID-19.

Five of the six hospitals had policies to test all pediatric patients for SARS-CoV-2 upon admission during the study period, allowing for detection of incidental positive SARS-CoV-2 test results. However, the proportion of such patients was smaller in this study compared with that in a previous report (*1*). Patients aged 0–4 and 12–17 years accounted for 79% of COVID-19–related hospitalizations in this study, which is consistent with data from other hospitals and communities (*2*). Among hospitalized children aged <5 years, most were aged <1 year, which might reflect clinical practice differences, because infants might be more likely to be hospitalized with milder disease than older children (*3*). Most patients were Black or Hispanic in this study; an earlier study demonstrated higher hospitalization rates among Black or Hispanic children compared with White children (*1*).

Approximately two thirds of patients hospitalized for COVID-19, including 83% and 88% of patients aged 5–11 and 12–17 years, respectively, had one or more underlying medical conditions. Approximately two thirds of patients hospitalized for COVID-19 aged 12–17 years had obesity. Compared with patients without obesity, those with obesity required higher levels and longer duration of care. These findings are consistent with previous reports (*4*) and highlight the importance of obesity and other medical conditions as risk factors for severe COVID-19 in children and adolescents.

The proportions of patients admitted to ICU and who required IMV are similar to those in prior reports, which predominantly included hospitalized pediatric COVID-19 patients before Delta variant predominance (*2*,*5*). Adolescents were more likely to require ICU admission and oxygen support compared with other age groups and required the longest median duration of IMV. The median duration of IMV overall (7 days) is consistent with previous reports (*6*,*7*). Approximately one half of patients aged 1–4 years required oxygen support, which might be related to the high proportion with viral coinfection. This study occurred during July–August 2021, the first period during the COVID-19 pandemic with high circulation of RSV^¶¶¶¶^ and other respiratory viruses. Compared with prior studies (*2*,*5*), this study found a high proportion of patients had high-flow nasal cannula as the highest level of respiratory support (37%), which might reflect a change in practice to avoid intubation or the high proportion of viral coinfections, including RSV.

On November 2, 2021, CDC recommended COVID-19 vaccinations for children aged 5–11 years (*8*). As of July 31, 2021, 29% of U.S. persons aged 12–17 years were fully vaccinated against COVID-19.***** In this study, only 0.4% of vaccine-eligible adolescents hospitalized for COVID-19 were fully vaccinated. Hospitalization rates have been shown to be 10 times higher among unvaccinated adolescents compared with fully vaccinated adolescents (*2*). Similarly, this study demonstrates that unvaccinated children hospitalized for COVID-19 could experience severe disease and reinforces the importance of vaccination of all eligible children to provide individual protection and to protect those who are not yet eligible to be vaccinated.

The findings in this report are subject to at least five limitations. First, the data came from only six hospitals, five of which are in the southern U.S. region. The proportion of adolescents with obesity in the southern United States is higher than in other regions,^†††††^ which might explain the high rates of obesity described in this report. Therefore, findings might not be generalizable to other areas. Second, findings might reflect differences in practices by hospitals or changes in practice over time and might not reflect differences in severity of COVID-19 related to the Delta variant. Third, incomplete or missing data in medical records might lead to underreporting and underestimation of details such as COVID-19 vaccination frequencies. Fourth, at the time of hospitalization, persons aged 12–15 years had only been vaccine-eligible for 2–3 months (*9*), possibly contributing to the low vaccination rates observed. Finally, hospitals identified patients for review based on positive polymerase chain reaction and antigen SARS-CoV-2 test results and hospitalization during the study period. Therefore, proportions of patients with MIS-C are likely underestimated.

Among pediatric patients with COVID-19–related hospitalizations, many had severe illness and viral coinfections, and few vaccine-eligible patients hospitalized for COVID-19 were vaccinated. These data highlight the importance of COVID-19 vaccination for those aged ≥5 years and other prevention strategies to protect children and adolescents from COVID-19, particularly those with obesity and other underlying health conditions. Further research and surveillance for viral coinfections with SARS-CoV-2 in pediatric patients can inform public health and capacity planning (*10*).

**Summary**

**What is already known about this topic?**

Pediatric COVID-19–related hospitalization rates increased when the highly transmissible SARS-CoV-2 B.1.617.2 (Delta) variant became the predominant circulating strain.

**What is added by this report?**

Among children and adolescents with SARS-CoV-2 infection admitted to six hospitals during July–August 2021, 77.9% were hospitalized for acute COVID-19. Among these patients, approximately one third aged <5 years had a viral coinfection (approximately two thirds of which were respiratory syncytial virus) and approximately two thirds of those aged 12–17 years had obesity; only 0.4% of age-eligible patients were fully vaccinated.

**What are the implications for public health practice?**

COVID-19 vaccination and other prevention strategies are important to protect children from COVID-19, particularly children with obesity and other underlying health conditions.

## Footnotes

* These authors contributed equally to this report.

† https://www.cdc.gov/coronavirus/2019-ncov/variants/delta-variant.html; https://covid.cdc.gov/covid-data-tracker/#variant-proportions (Accessed September 15, 2021).

§
https://covid.cdc.gov/covid-data-tracker/#new-hospital-admissions

¶
https://www.cdc.gov/mmwr/volumes/70/wr/mm7020e1.htm?s_cid=mm7020e1_w

**
https://www.cdc.gov/mmwr/volumes/70/wr/mm7045e1.htm?s_cid=mm7045e1_w

†† COVID-19 was confirmed with laboratory detection of SARS-CoV-2 by reverse transcription–polymerase chain reaction or antigen test.

§§ Patients with MIS-C as the reason for hospitalization included patients who met the clinical case definition for MIS-C (clinically severe illness requiring hospitalization in a person aged <21 years with fever, laboratory evidence of inflammation, multisystem [≥2] organ involvement and no alternative plausible diagnosis, and evidence of current or recent SARS-CoV-2 infection by reverse transcription polymerase chain reaction, serology or antigen test, or COVID-19 exposure within the 4 weeks preceding symptom onset [https://emergency.cdc.gov/han/2020/han00432.asp]) and were hospitalized for diagnosis and management of MIS-C, based on chart review.

¶¶ Patients were considered to have a viral coinfection if they had ≥1 of the following infections: type A influenza, type B influenza, unspecified influenza, coronavirus 229e, coronavirus hku1, coronavirus nl63, coronavirus 0c43, respiratory syncytial virus, adenovirus, parainfluenza type 1, parainfluenza type 2, parainfluenza type 3, parainfluenza type 4, human metapneumovirus, rhinovirus, enterovirus, or other viral coinfection.

*** Fully vaccinated was defined as having received 2 doses of an mRNA-based COVID-19 vaccine ≥14 days before hospital admission date. Partially vaccinated was defined as having received only 1 dose of an mRNA-based COVID-19 vaccine ≥14 days before hospitalization. All vaccinated patients in this study received the Pfizer-BioNTech (BNT162b2) vaccine.

††† A convenience sample of six hospitals was selected among members of the Children’s Hospital Association. All hospitals were in jurisdictions with a high level of COVID-19 community transmission during July–August 2021; these jurisdictions were not represented by the COVID-NET surveillance system. https://www.cdc.gov/coronavirus/2019-ncov/covid-data/covid-net/purpose-methods.html

§§§ COVID-19 diagnosis indicated in medical record or based on positive SARS-CoV-2 test result (antigen or polymerase chain reaction/nucleic acid amplification test, or antibody test among patients with a diagnosis of MIS-C).

¶¶¶ Abstractors selected the category that best fit the overall reason for hospitalization, with adjudication by project leaders, and through audits of 5% of all charts.

**** ECMO is a form of advanced life support used in patients with medically refractory respiratory or cardiac failure.

†††† For children aged ≥2 years, height and weight were used to calculate body mass index (BMI) (kg/m^2^). BMI percentiles were calculated using BMI, age, and sex. Children with BMI percentiles ≥95% were considered to have obesity (https://www.cdc.gov/obesity/childhood/defining.html) and those with BMI ≥120% of the 95th percentile were considered to have severe obesity. BMI data extracted from charts was used if height or weight was missing. If BMI was missing or unable to be calculated, a diagnosis of obesity recorded in charts was used and severity of obesity was unable to be assessed. Obesity was not assessed for children aged <2 years.

§§§§ 45 C.F.R. part 46, 21 C.F.R. part 56; 42 U.S.C. Sect. 241(d); 5 U.S.C. Sect. 552a; 44 U.S.C. Sect. 3501 et seq.

¶¶¶¶ https://emergency.cdc.gov/han/2021/han00443.asp; https://www.cdc.gov/surveillance/nrevss/rsv/natl-trend.html

*****
https://covid.cdc.gov/covid-data-tracker/#vaccination-demographics-trends

†††††
https://nccd.cdc.gov/dnpao_dtm/rdPage.aspx?rdReport=DNPAO_DTM.ExploreByTopic&islClass=OWS&islTopic=&go=GO
