# *Erratum:* Vol. 70, No. 51–52

**DOI:** 10.15585/mmwr.mm7105a5

**Published:** 2022-02-04

In the report, “Characteristics and Clinical Outcomes of Children and Adolescents Aged <18 Years Hospitalized with COVID-19 — Six Hospitals, United States, July–August 2021,” on page 1766, the list of authors should have read,

“Valentine Wanga, PhD^1,2^; Megan E. Gerdes, MPH^1^; Dallas S. Shi, MD, PhD^1,2^; Rewa Choudhary, MD^1,2^; Theresa M. Dulski, MD^1,2^; Sophia Hsu, MSN, MPH^1^; Osatohamwen I. Idubor, MD^1^; Bryant J. Webber, MD^1,2^; Arthur M. Wendel, MD^1^; Nickolas T. Agathis, MD^1,2^; Kristi Anderson, MD^1^; Tricia Boyles, MHA^1^; Sophia K. Chiu, MD^1^; Eleanor S. Click, MD, PhD^1^; Juliana Da Silva, MD^1^; Hannah Dupont, MPH^1^; Mary Evans, MD^1^; Jeremy A.W. Gold, MD^1^; Julia Haston, MD^1,2^; Pamela Logan, MD^1^; Susan A. Maloney, MD^1^; Marisol Martinez, PharmD^1^; Pavithra Natarajan, BMBS^1^; Kevin B. Spicer, MD, PhD^1^; Mark Swancutt, MD^1^; Valerie A. Stevens^1^; Jessica Brown, PhD^1^; Gyan Chandra, MBA^1^; Megan Light, MPH^1^; Frederick E. Barr, MD^3^; Jessica Snowden, MD^3^; Larry K. Kociolek, MD^4^; Matthew McHugh, MPH^4^; David Wessel, MD^5^; Joelle N. Simpson, MD^5^; Kathleen C. Gorman, MSN^5^; Kristen A. Breslin, MD^5^; Roberta L. DeBiasi, MD^5^; Aaron Thompson, MD^6,7^; Mark W. Kline, MD^6,7^; **Julie A. Boom, MD^8,10^**; **Ila R. Singh, MD, PhD^9,10^**; Michael Dowlin^9^; **Mark Wietecha, MS, MBA^11^**; Beth Schweitzer, MS^1^; Sapna Bamrah Morris, MD^1^; Emily H. Koumans, MD^1^; Jean Y. Ko, PhD^1^; Anne A. Kimball, MD^1,^*; David A. Siegel, MD^1,^*”

In addition, on page 1771, the list of author affiliations should have read,

^“1^CDC COVID-19 **Emergency** Response Team; ^2^Epidemic Intelligence Service, CDC; ^3^Arkansas Children’s, Little Rock, Arkansas; ^4^Ann & Robert H. Lurie Children's Hospital of Chicago, Chicago, Illinois; ^5^Children’s National Hospital, Washington, DC; ^6^Children’s Hospital New Orleans, New Orleans, Louisiana; ^7^Tulane University School of Medicine and LSU Health, New Orleans, Louisiana; ^8^Department of Pediatrics, Baylor College of Medicine, Houston, Texas; ^9^Department of Pathology and Immunology, Baylor College of Medicine, Houston, Texas; **^10^Texas Children’s Hospital, Houston, Texas**; **^11^Children’s Hospital Association, Washington, DC**.”

